# Supplementary material for: Adjuvant Effects of a New Saponin Analog VSA-1 on Enhancing Homologous and Heterosubtypic Protection by Influenza Virus Vaccination
Source: Vaccines (Basel). 2022 Aug 24;10(9):1383. doi: 10.3390/vaccines10091383 (PMC9501088; doi:10.3390/vaccines10091383)
Supplement: Supplementary file 1 [file vaccines-10-01383-s001.zip › vaccines-1815682-SM.pdf]

Supplementary Information for

Adjuvant Effects of a New Saponin Analog VSA-1 on Enhancing Homologous and Heterosubtypic Protection by Influenza Virus Vaccination

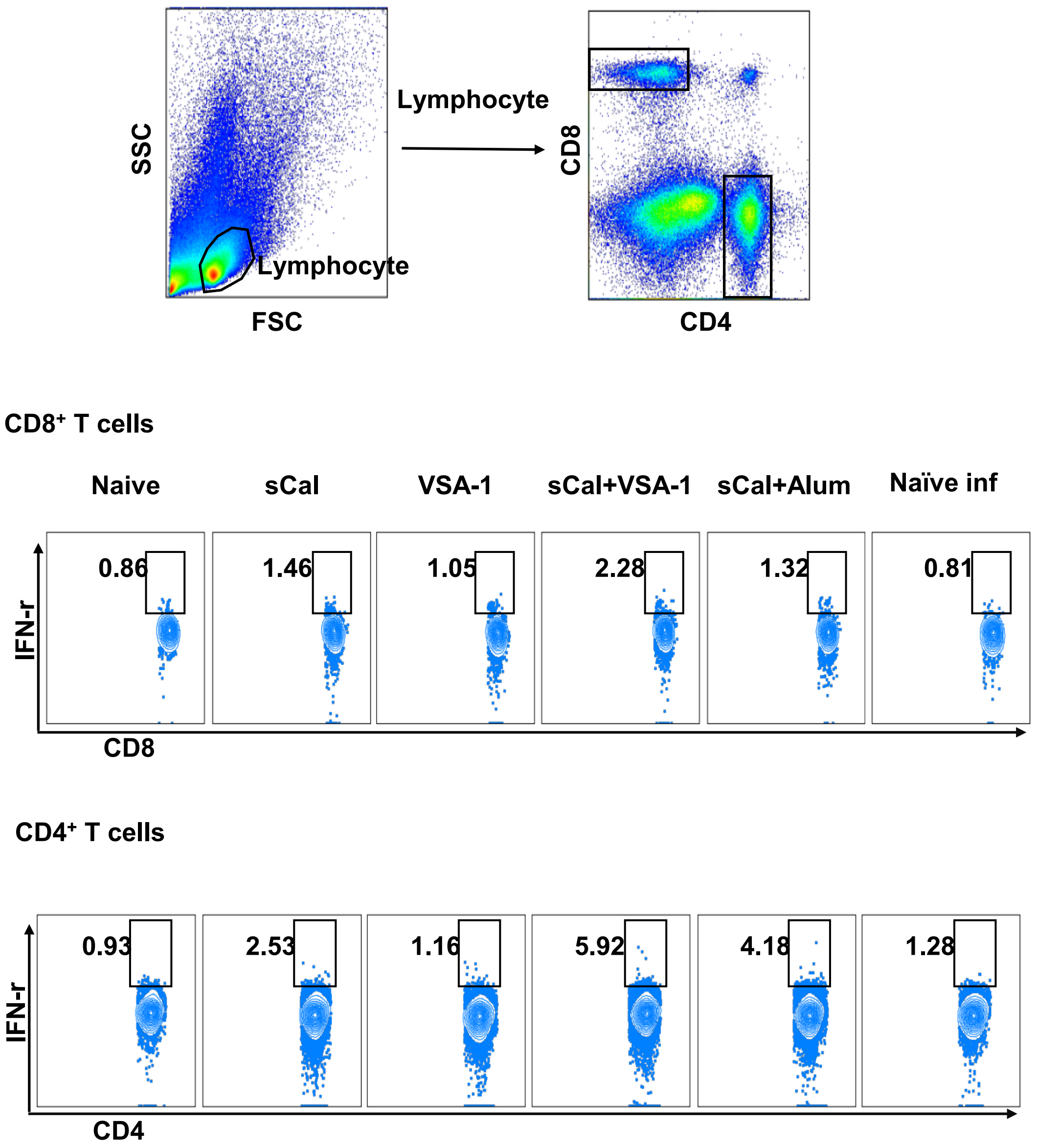

Supplementary Figure S1. The gating strategies and representative flow cytometry data of each group of mice. The total CD4 and CD8 T cells were gated from the lymphocytes of the first gated cell populations. The IFN-γ positive cell populations were further gated from the total CD4 and CD8 T cells gated, and then presented in percentages out of CD4 or CD8 T cells.

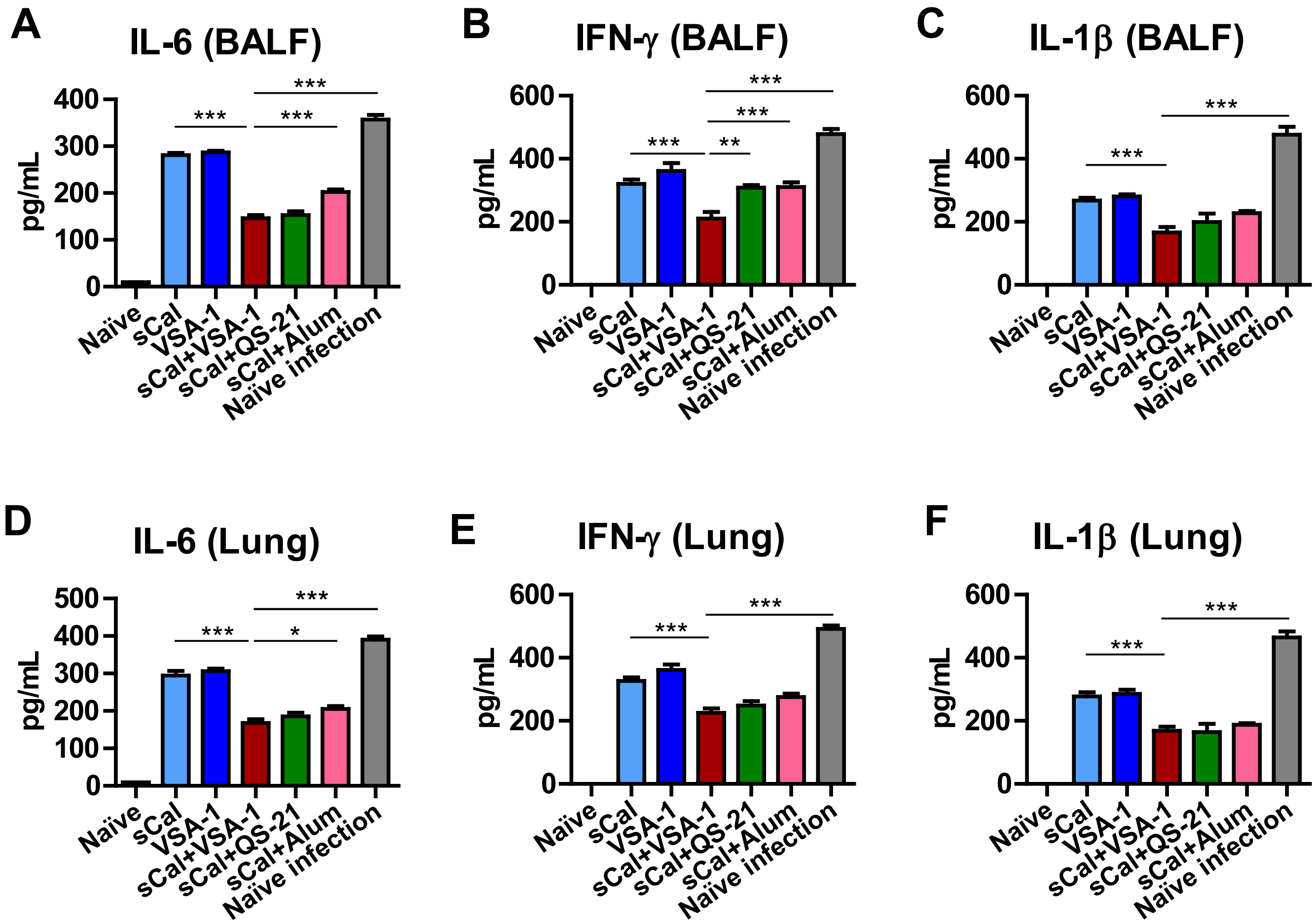

Supplementary Figure S2. Cytokines in BALF and lungs of mice with prime vaccination after homologous virus challenge. C57BL/6 mice (n = 3 per group) were intramuscularly immunized with sCal (3 µg) split vaccine alone or sCal plus VSA-1 (50 µg), QS-21 (10 µg) or Alum (50 µg). VSA-1 (50 µg) only immunized mice were used as mock control. Three to four weeks post immunization, the mice were challenged with a lethal dose of A/Cal H1N1 virus (~~2~~3 × LD_{50}). BALF and lung samples of the immunized C57BL/6 mice (n = 3 per group) were harvested at day 5 post A/Cal H1N1virus infection. Cytokine levels of BALF **(A-C)** and lung extracts **(D-F)** were measured by ELISA. Statistical significance was calculated by using one-way ANOVA and Dunnett's post-multiple comparison tests. Error bars indicate the mean ± standard errors of the mean (SEM). *; $p < 0.05$, **; $p < 0.01$, ***; $p < 0.001$ compared to the sCal+VSA-1 group.

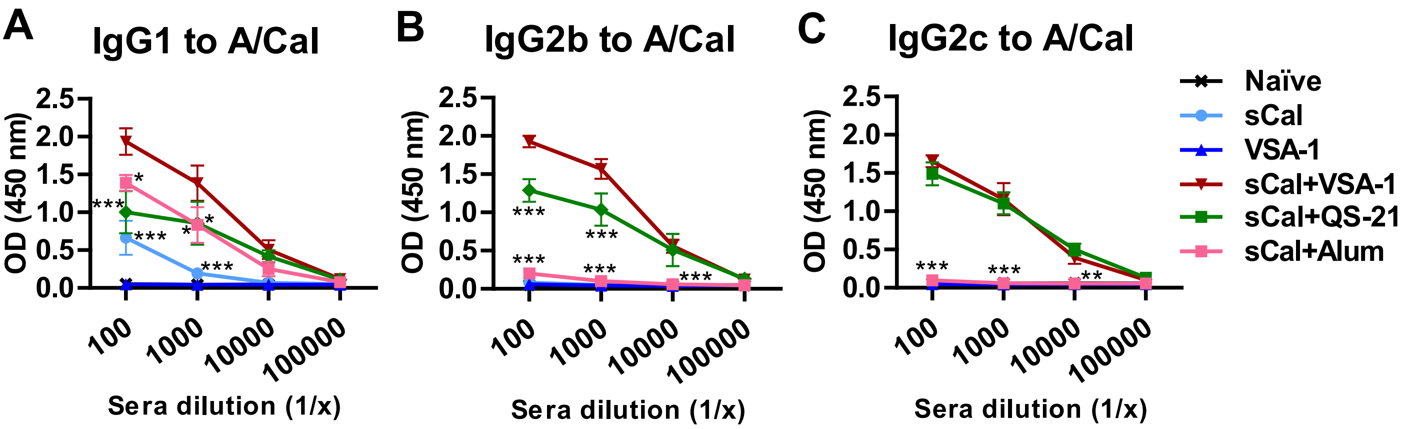

Supplementary Figure S3. VSA-1 adjuvanted prime-boost influenza vaccination enhances virus-specific IgG isotype switched antibodies in C57BL/6 mice. C57BL/6 mice (n = 3 per group) were intramuscularly prime-boost immunized with sCal (3 µg) split vaccine alone or sCal plus VSA-1 (50 µg prime, 25 µg boost), QS-21 (10 µg) or Alum (50 µg). VSA-1 (50 µg prime, 25 µg boost) only immunized mice were used as mock control. **(A-C)** A/Cal H1N1 virus-specific IgG isotype antibody levels in boost sera. Error bars indicate the mean ± standard errors of the mean (SEM). *; $p < 0.05$, **; $p < 0.01$, ***; $p < 0.001$ compared to the sCal+VSA-1 group.

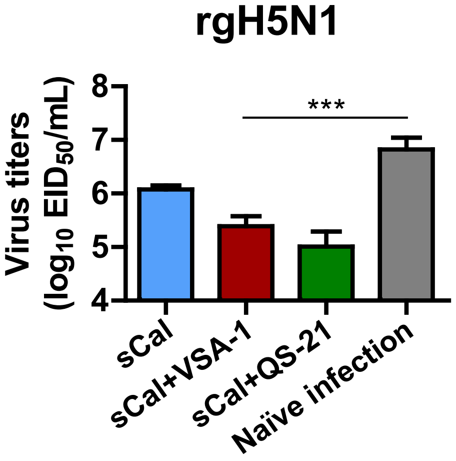

Supplementary Figure S4. VSA-1 adjuvanted prime-boost influenza vaccination controls lung viral loads in C57BL/6 mice. C57BL/6 mice (n = 3 per group) were intramuscularly prime-boost immunized with sCal (3 µg) split vaccine alone or sCal plus VSA-1 (50 µg prime, 25 µg boost) or QS-21 (10 µg). Lung samples of the immunized C57BL/6 mice were harvested at day 6 post rgH5N1 virus infection. Error bars indicate the mean ± standard errors of the mean (SEM). *; $p < 0.05$, **; $p < 0.01$, ***; $p < 0.001$ compared to the sCal+VSA-1 group.

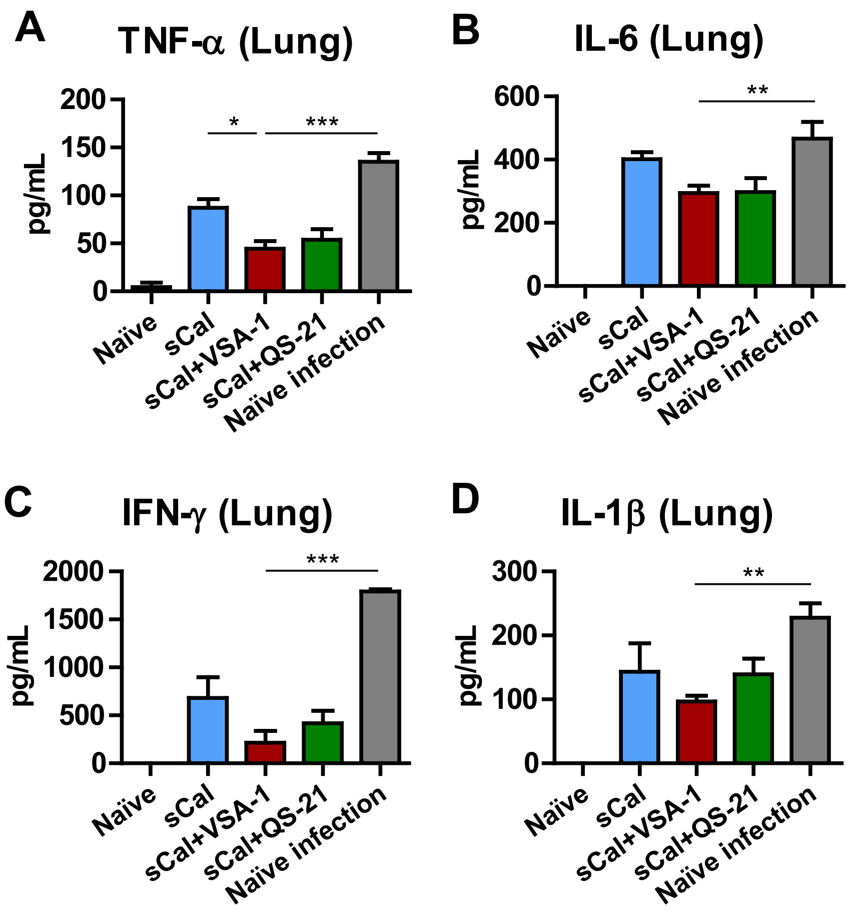

Supplementary Figure S5. VSA-1 adjuvanted prime-boost influenza vaccination prevents the induction of inflammatory cytokines after lethal challenge with heterosubtypic rgH5N1 virus in C57BL/6 mice. Vaccination and challenge regimen is the same as described in Supplementary Figure S4. Lung samples of the immunized C57BL/6 mice were harvested at day 6 post rgH5N1 virus infection. **(A)** TNF-α, **(B)** IL-6, **(C)** IFN-γ, and **(D)** IL-1β cytokine levels of lung extracts were measured by ELISA. Error bars indicate the mean ± standard errors of the mean (SEM). *; $p < 0.05$, **; $p < 0.01$, ***; $p < 0.001$ compared to the sCal+VSA-1 group.

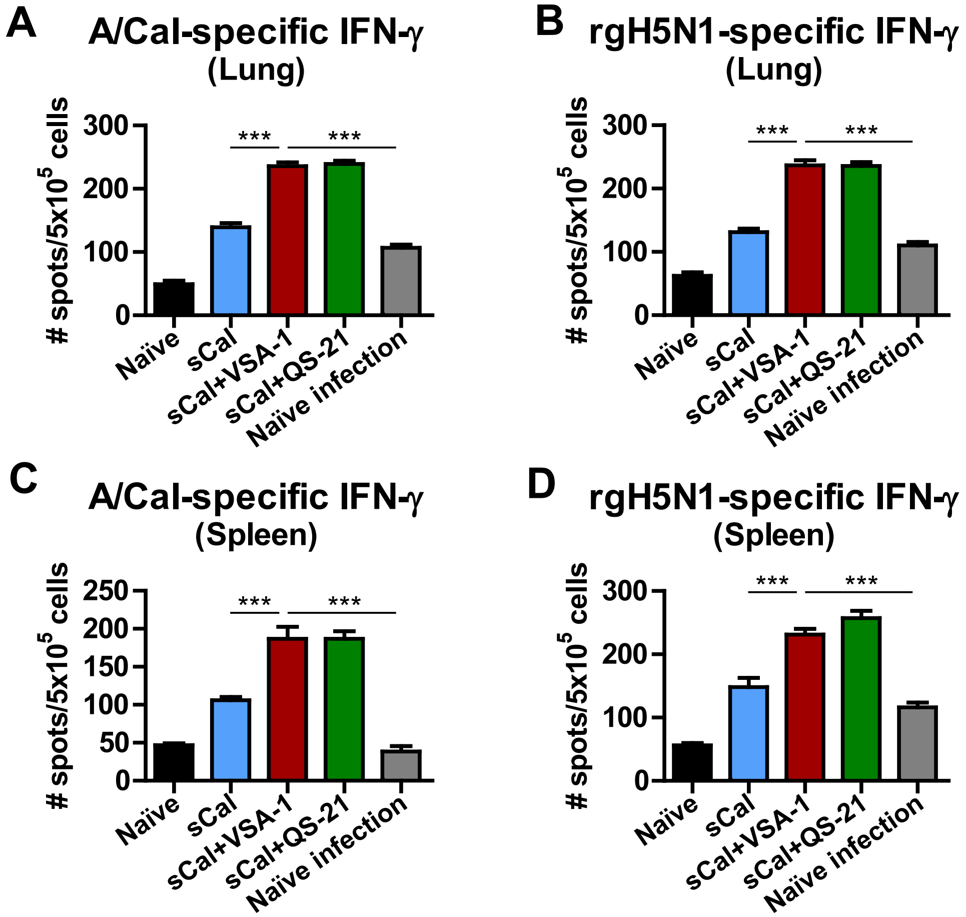

Supplementary Figure S6. VSA-1 adjuvanted prime-boost influenza vaccination enhances effector T cells after lethal challenge with heterosubtypic rgH5N1 virus in C57BL/6 mice. Vaccination and challenge regimen is the same as described in Supplementary Figure S4. Lung and spleen samples of the immunized C57BL/6 mice were harvested at day 6 post rgH5N1 virus infection. **(A-D)** Cytokine ELISpot of lung cells **(A and B)** and splenocytes **(C and D)** at day 6 post infection after in vitro stimulation with inactivated A/Cal H1N1 virus or rgH5N1 virus. Error bars indicate the mean ± standard errors of the mean (SEM). ***; $p < 0.001$ compared to the sCal+VSA-1 group.
